# Egr-1 and RNA POL II facilitate glioma cell GDNF transcription induced by histone hyperacetylation in promoter II

**DOI:** 10.18632/oncotarget.15126

**Published:** 2017-02-06

**Authors:** Bao-Le Zhang, Ting-Wen Guo, Le-Le Gao, Guang-Quan Ji, Xiao-He Gu, Yu-Qi Shao, Rui-Qin Yao, Dian-Shuai Gao

**Affiliations:** ^1^ Department of Neurobiology and Anatomy, Xuzhou Key Laboratory of Neurobiology, Jiangsu Key Laboratory of New Drug Research and Clinical Pharmacy, Xuzhou Medical University, Xuzhou 221004, Jiangsu, China

**Keywords:** Egr-1, GDNF, RNA POL II, histone acetylation, glioma

## Abstract

The specific mechanisms for epigenetic regulation of gene transcription remain to be elucidated. We previously demonstrated that hyperacetylation of histone H3K9 in promoter II of glioma cells promotes high transcription of the glial cell line-derived neurotrophic factor (*GDNF*) gene. This hyperacetylation significantly enhanced Egr-1 binding and increased the recruitment of RNA polymerase II (RNA POL II) to that region (*P* < 0.05). Egr-1 expression was abnormally increased in C6 glioma cells. Further overexpression of Egr-1 significantly increased Egr-1 binding to *GDNF* promoter II, while increasing RNA POL II recruitment, thus increasing *GDNF* transcription (*P* < 0.01). When the acetylation of H3K9 in the Egr-1 binding site was significantly reduced by the histone acetyltransferase (HAT) inhibitor curcumin, binding of Egr-1 to *GDNF* promoter II, RNA POL II recruitment, and *GDNF* mRNA expression were significantly downregulated (*P* < 0.01). Moreover, curcumin attenuated the effects of Egr-1 overexpression on Egr-1 binding, RNA POL II recruitment, and *GDNF* transcription (*P* < 0.01). Egr-1 and RNA POL II co-existed in the nucleus of C6 glioma cells, with overlapping regions, but they were not bound to each other. In conclusion, highly expressed Egr-1 may be involved in the recruitment of RNA POL II in *GDNF* promoter II in a non-binding manner, and thereby involved in regulating *GDNF* transcription in high-grade glioma cells. This regulation is dependent on histone hyperacetylation in *GDNF* promoter II.

## INTRODUCTION

Glial cell line-derived neurotrophic factor (GDNF) is a member of the transforming growth factor (TGF-β) superfamily and an important neurotrophic factor. It was initially cloned from the rat B49 glial cell line [1]. In human cells, *GDNF* is a single-copy gene with two promoters and six exons located on chromosome 5 at p12-p13.1 [2, 3]. As it is a powerful factor promoting glioma cell proliferation and migration, GDNF is closely related to glioma development [4–8]. Its transcription is significantly higher in glioma cells than in normal glial cells [9, 10]. We previously reported that abnormal high-level *GDNF* transcription in glioma cells was related to histone hyperacetylation in its promoter II rather than gene mutation [11]. A subsequent study showed three consecutive and highly conserved binding sites for early growth response protein-1 (Egr-1) were located −186 bp upstream of the transcription initiation site in rat *GDNF* promoter II [12]. Histone hyperacetylation at these sites facilitates high-level *GDNF* transcription in C6 glioma cells [13], but the specific mechanism has not yet been elucidated.

The transcription factor Egr-1 has been shown to regulate cell growth, differentiation, and tumor progression [14–16]. It is highly expressed in tumor tissues such as liver and prostate cancer tissues [17, 18]. Moreover, Egr-1 overexpression has been shown to significantly promote the infiltration and migration of high-grade glioma cells [19, 20], which is consistent with the biological effect induced by the high-level *GDNF* transcription [4, 7]. We therefore hypothesized that Egr-1 expression might also be abnormally increased in high-grade glioma cells and that this transcription factor could mediate histone hyperacetylation-induced high-level *GDNF* transcription. In addition, bioinformatics analysis revealed that the upstream sequence of the *GDNF* gene transcription initiation site was rich in CG bases. Moreover, no typical TATA-box motif was found from −25 to −30 bp, which is to say *GDNF* promoter II region is TATA-less. CG-rich TATA-less promoters are thought to recruit RNA polymerase II (RNA POL II) by increasing their binding to gene-specific transcription factors, thereby initiating or promoting gene transcription [21]. Three consecutive and conserved Egr-1 binding sites are located in the CG-rich region upstream of the *GDNF* promoter II [13]. Therefore, we further hypothesized that Egr-1 might be involved in histone hyperacetylation-mediated *GDNF* transcription in high-grade glioma cells by increasing RNA POL II recruitment to the Egr-1 binding sites of *GDNF* promoter II.

We examined acetylation of histone H3 Lys9 (H3K9) in the Egr-1 binding sites of *GDNF* promoter II, as well as their binding to Egr-1 and RNA POL II, in normal and diseased human tissue by chromatin immunoprecipitation (ChIP). Subsequently, the expression of Egr-1 in human high-grade and low-grade glioma tissues, normal brain tissue, and rat C6 astroglioma cells and normal astrocytes were measured by real-time polymerase chain reaction (PCR) and western blot. Next, the effects of Egr-1 overexpression on Egr-1 binding to *GDNF* promoter II, RNA POL II recruitment, and *GDNF* transcription in C6 glioma cells were determined by gene overexpression, ChIP, and real-time PCR. The effects of Egr-1 overexpression on these outcomes were elucidated using a C6 glioma cell model with decreased histone acetylation in *GDNF* promoter II following treatment with the histone acetyltransferase (HAT) inhibitor curcumin [22–24]. Finally, the presence and mutual binding of RNA POL II and Egr-1 in the nuclei of C6 astroglioma cells were assessed by immunofluorescence and co-immunoprecipitation (co-IP). Our findings provide new clues to the mechanism underlying abnormally high transcription of *GDNF* in glioma cells.

## RESULTS

### Increased H3K9 acetylation in Egr-1 binding sites of *GDNF* promoter II in high-grade glioma tissue

**Figure 1:**
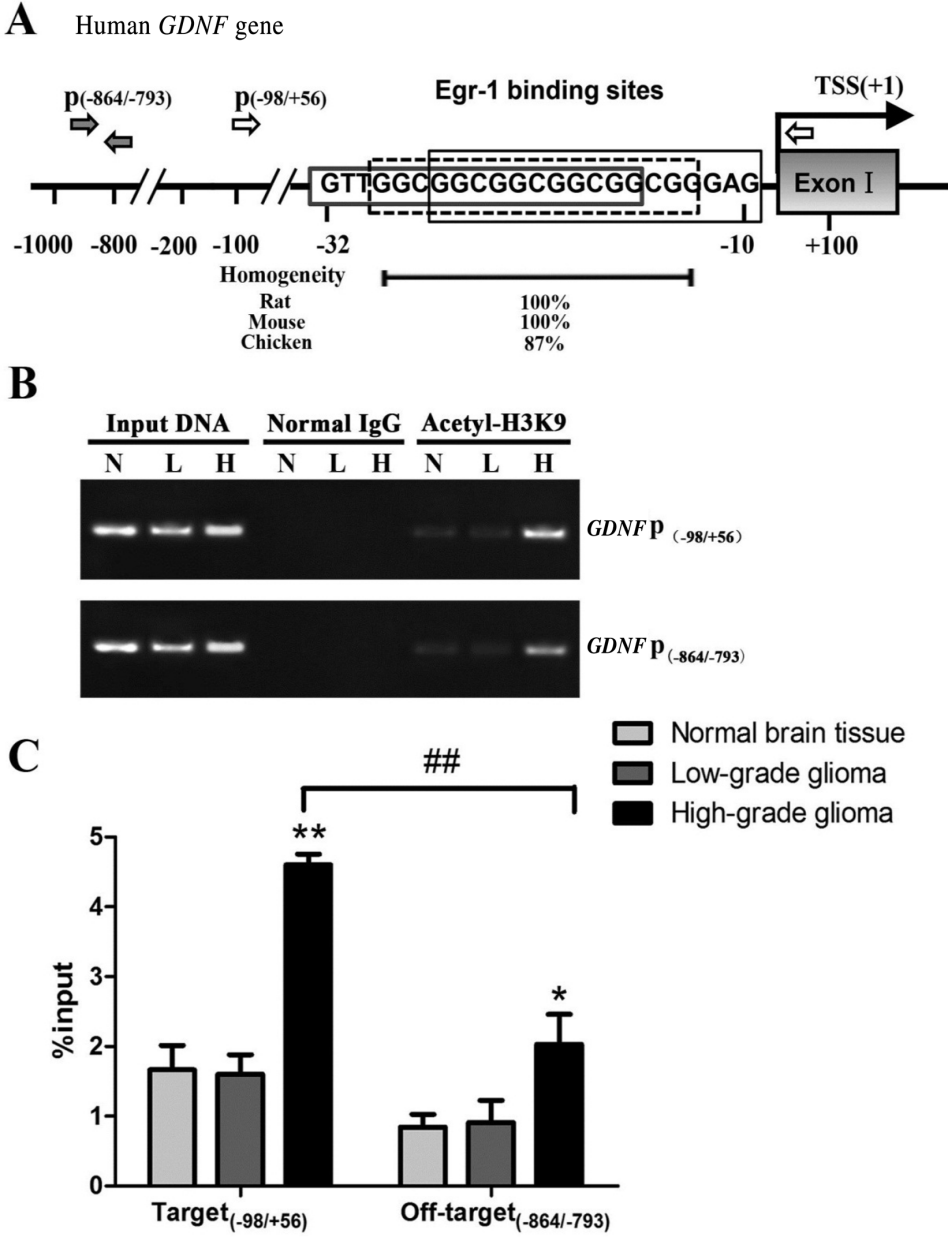
H3K9 acetylation of *GDNF* gene promoter region II in human high-grade glioma tissue (H), low-grade glioma tissue (L), and normal brain tissue (N) (**A**) Schematic showing the Egr-1 binding site in human *GDNF* gene promoter region II. The black and white arrows indicate the ChIP-PCR primer positions. The transcription start site (TSS) of the promoter II-derived transcript is +1. Phylogenetic conservation values are indicated. (**B**) ChIP analyses with anti-acetyl-histone H3 (Lys9) antibody or normal rabbit IgG (negative control). Immunoprecipitated and input DNA were assayed using PCR with specific primers (P(−98/+56), P(−864/−793)). (**C**) The ChIP DNA value was standardized with input DNA, and the data are the percentages of input chromatin. *P < 0.05, **P < 0.01, ^##^*P* < 0.01.

Three consecutive and conserved Egr-1 binding sites were present −32 bp upstream of the transcription initiation site in human *GDNF* promoter II (Figure 1A). H3K9 acetylation in the Egr-1 binding sites (on-target region −98/+56 bp) and off-target region (−864/−793 bp) of *GDNF* promoter II was measured in high-grade and low-grade glioma tissues and normal brain tissue using ChIP-PCR. The primer positions are shown in Figure 1A. The histone H3K9 in the Egr-1 binding sites of *GDNF* promoter II was acetylated to varying degrees (Figure 1B). Acetylation was significantly higher in the Egr-1 on-target region of *GDNF* promoter II (*P* < 0.01) and the off-target region (*P* < 0.05) in high-grade glioma tissue compared with normal brain tissue. Moreover, the degree of acetylation was significantly higher in the Egr-1 on-target region than in the off-target region (*P* < 0.01). This is consistent with our previous report of a difference in H3K9 acetylation in *GDNF* promoter II of rat C6 glioma cells [13]. By contrast, there was no significant difference in H3K9 acetylation in the Egr-1 on- and off-target regions in low-grade glioma tissue compared with normal brain tissue (*P* > 0.05, Figure 1C).

### Increased Egr-1 binding to *GDNF* promoter II in high-grade glioma tissue

**Figure 2:**
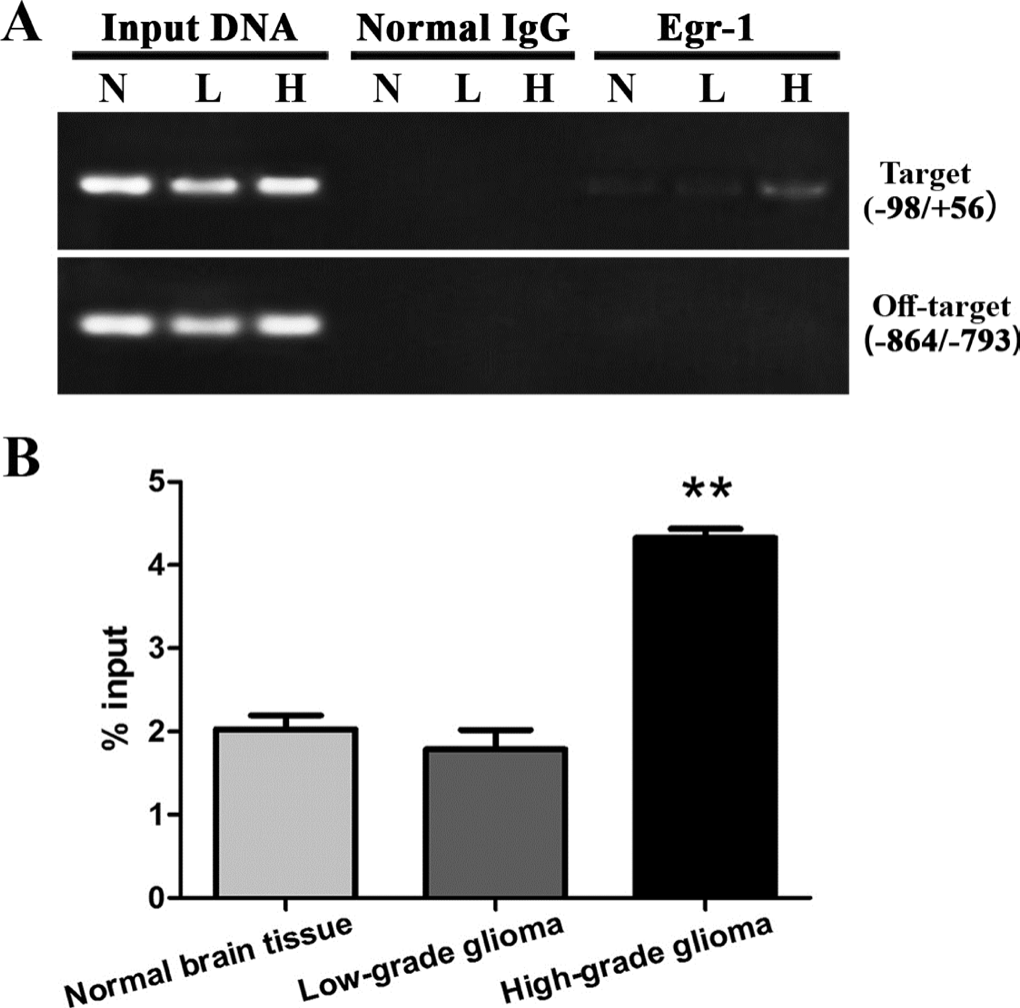
Egr-1 binding to *GDNF* gene promoter region II in human high-grade glioma tissue (H), low-grade glioma tissue (L), and normal brain tissue (N) (**A**) An anti-Egr-1 antibody or normal rabbit IgG was used for ChIP analysis following cross-linking and chromatin breakage. Specific primers for target P(−98/+56) and off-target P(−864/−793) areas and immunoprecipitated DNA and input DNA were detected with PCR. Specific primers for target area P(−98/+56) amplified target fragments to variable degrees, but using the same ChIP DNA template, the primer for the off-target area P(−864/−793) could not produce target fragments with PCR amplification, while target fragments were procured using input DNA. (**B**) After real-time PCR, ChIP DNA values were standardized with input DNA, and data are the percentages of chromatin. **P < 0.01.

Egr-1 binding to *GDNF* promoter II was measured by ChIP-PCR. The results showed that Egr-1 could bind between −98 and +56 of *GDNF* promoter II in all three types of tissues but not to the off-target region (between −864 and −793). This demonstrated the specific binding of Egr-1 to the Egr-1 binding sites of *GDNF* promoter II (Figure 2A). Compared with normal brain tissue, Egr-1 binding was very significantly increased in high-grade glioma tissue (*P* < 0.01). This is consistent with the difference in H3K9 acetylation in *GDNF* promoter II of rat C6 glioma cells we previously reported [13]. There was no significant difference between low-grade glioma tissue and normal brain tissue (*P* > 0.05, Figure 2B).

### Increased recruitment of RNA POL II to the Egr-1 binding sites of *GDNF* promoter II in high-grade glioma tissue and cells

**Figure 3:**
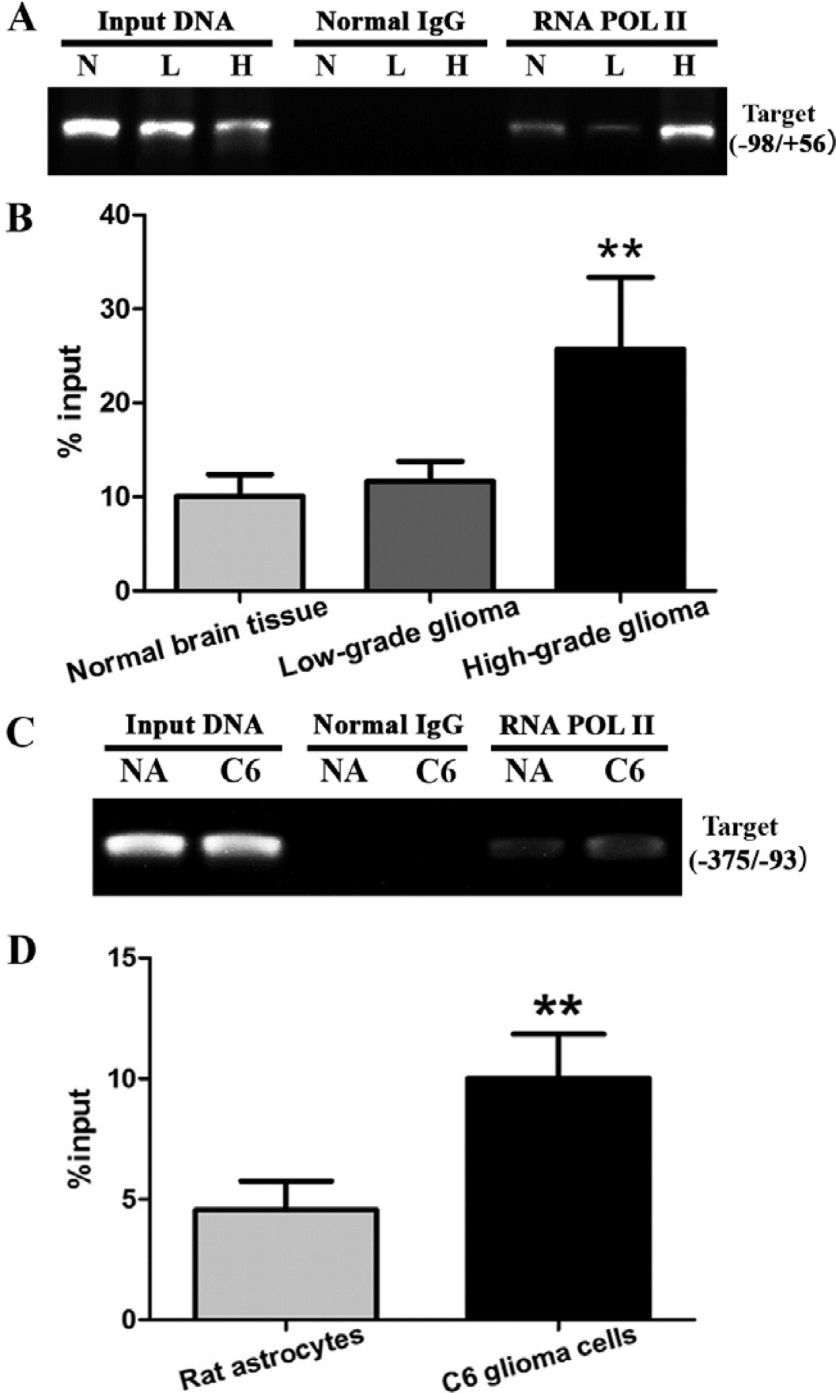
RNA POL II recruitment to Egr-1 binding sites of *GDNF* promoter II in glioma tissues and cells (**A, B**) RNA POL II recruitment was measured in different grades of glioma tissues using ChIP-PCR. (**C, D**) Recruitment was measured in rat C6 astroglioma cells and normal astrocytes using ChIP-PCR. **P < 0.01.

RNA POL II binding to the Egr-1 binding sites of *GDNF* promoter II was measured by ChIP-PCR to assess the recruitment of RNA POL II in the Egr-1 binding sites of *GDNF* promoter II in different grades of glioma. The results showed that RNA POL II could bind to different extents in all three types of tissues (Figure 3A). Compared with normal brain tissue, RNA POL II recruitment to the Egr-1 binding sites was significantly increased in high-grade glioma tissue (*P* < 0.05). In contrast, there was no significant difference between low-grade glioma tissue and normal brain tissue (*P* > 0.05, Figure 3B). To further determine whether this phenomenon occurs in high-grade glioma cells, RNA POL II recruitment to Egr-1 binding sites (between −375 and −93) of rat *GDNF* promoter II was measured in rat C6 astroglioma cells and normal astrocytes using ChIP-PCR. The results showed that RNA POL II recruitment was also significantly increased in C6 astroglioma cells (*P* < 0.01, Figure 3C and 3D).

### Increased Egr-1 expression in high-grade glioma tissue and cells

**Figure 4:**
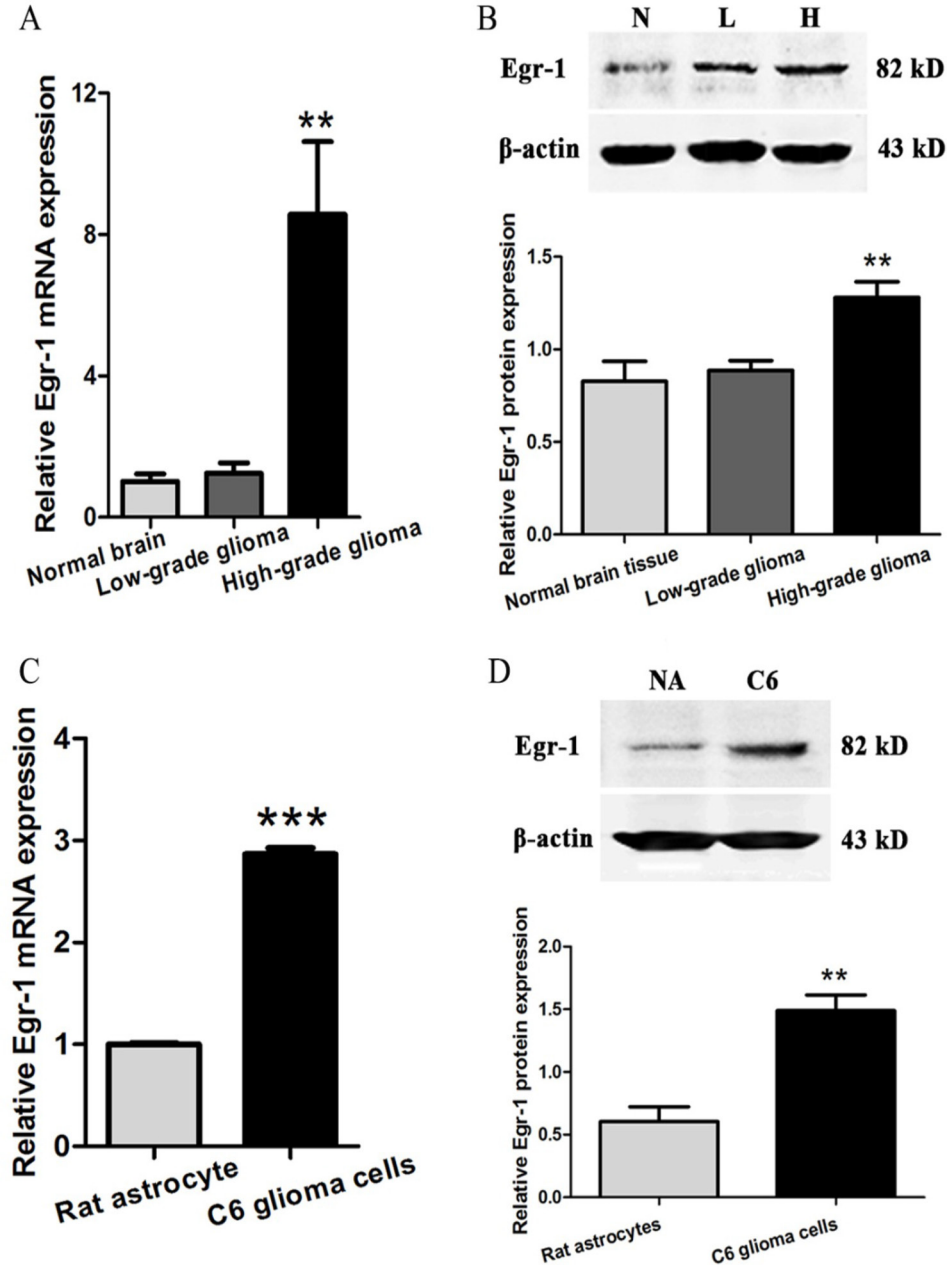
Egr-1 expression levels in glioma tissues and cells (**A, B**) Relative expression of Egr-1 mRNA and protein in high-grade glioma tissue (H), low-grade glioma tissue (L) and normal brain tissue (N). (**C, D**) Relative Egr-1 mRNA and protein expression in rat C6 astroglioma cells and rat normal astrocytes (NA). β-actin was used as the internal reference protein. **P < 0.01, ***P < 0.001.

Egr-1 expression was measured in high-grade and low-grade glioma tissues and normal brain tissue using real-time PCR and western blot. The results showed that mRNA and protein expression of Egr-1 in high-grade glioma tissue were significantly higher than that in normal brain tissue (*P* < 0.01). In contrast, there was no significant difference between low-grade glioma and normal brain tissues (*P* > 0.05, Figure 4A and 4B). To further determine whether this phenomenon is also present in high-grade glioma cells, Egr-1 expression was measured in rat C6 astroglioma cells and normal astrocytes using real-time PCR and western blot. The results showed that the mRNA and protein expressions of Egr-1 in C6 glioma cells were also significantly higher than that in normal astrocytes (*P* < 0.01, Figure 4C and 4D).

### Egr-1 overexpression significantly increased RNA POL II recruitment to *GDNF* promoter II and *GDNF* transcription in C6 glioma cells

**Figure 5:**
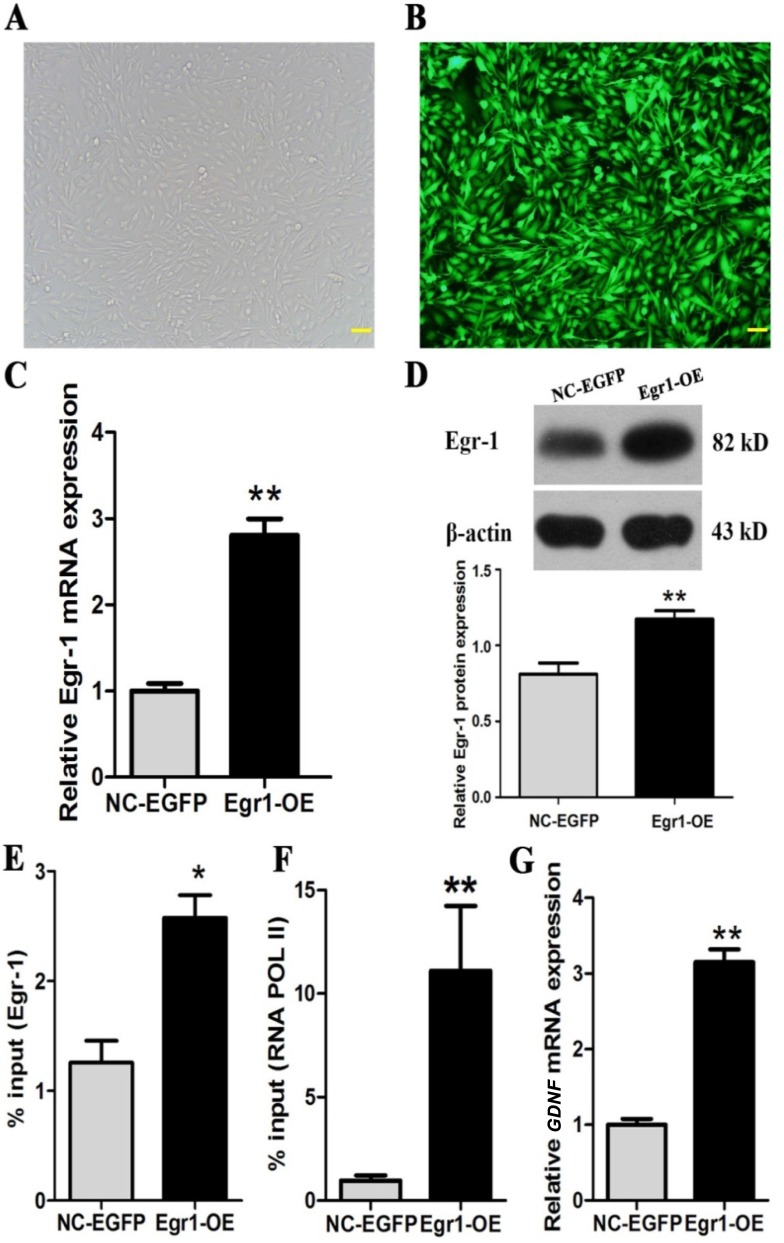
Egr-1 overexpression significantly increased Egr-1 binding to *GDNF* promoter II, RNA POL II recruitment, and *GDNF* transcription in rat C6 glioma cells C6 glioma cells in the logarithmic growth phase were inoculated into 6-well plates and then infected with 100 MOI of lentivirus Egr1-OE and lentivirus NC-EGFP when the cells reached 70% confluence. After 72 h, the virus infection rate was observed under the fluorescence microscope. (**A, B**) More than 90% of C6 glioma cells had normal morphology and showed green fluorescence. Real-time PCR, western blot, and ChIP-PCR showed that (**C**) Egr-1 mRNA and (**D**) protein expression significantly increased in C6 cells infected with lentivirus Egr1-OE (*P* < 0.01). (**E**) Egr-1 binding to *GDNF* promoter II, (**F**) RNA POL II recruitment in that region, and (**G**) *GDNF* mRNA expression were also increased significantly (*P* < 0.05). The results are from three independent experiments. *P < 0.05, **P < 0.01. The scale is 100 μm.

To investigate the relationship of Egr-1 overexpression with RNA POL II recruitment to *GDNF* promoter II and *GDNF* transcription in high-grade glioma cells, C6 astroglioma cells were infected with 100 MOI of lentivirus Egr1-OE. The infection rate was then determined 72 h later with fluorescence microscopy. Egr- 1 overexpression was examined by real-time PCR and western blot. The effects of Egr-1 overexpression on the binding of Egr-1 and RNA POL II to *GDNF* promoter II and *GDNF* transcription were examined by ChIP-PCR and real-time PCR in C6 astroglioma cells. The results showed that the infection efficiency of lentivirus Egr1-OE was > 90%, and the cells were in good condition after infection (Figure 5A and 5B). Both mRNA (Figure 5C) and protein (Figure 5D) expression of Egr-1 were significantly increased (*P* < 0.01) after infection, indicating the successful establishment of a C6 glioma cell model with overexpressed Egr-1. Furthermore, Egr-1 overexpression in C6 astroglioma cells significantly increased both binding of Egr-1 to the Egr-1 binding sites of *GDNF* promoter II (*P* < 0.05, Figure 5E) and recruitment of RNA POL II in that region (Figure 5F). *GDNF* transcription was also significantly enhanced (*P* < 0.01, Figure 5G). Notably, the results showed that Egr-1 protein expression had the most significant positive correlation with *GDN*F transcription (r = 0.974, *P* < 0.01).

### Histone hypoacetylation in the Egr-1 binding sites of *GDNF* promoter II inhibited *GDNF* transcription induced by Egr-1 overexpression in C6 glioma cells

**Figure 6:**
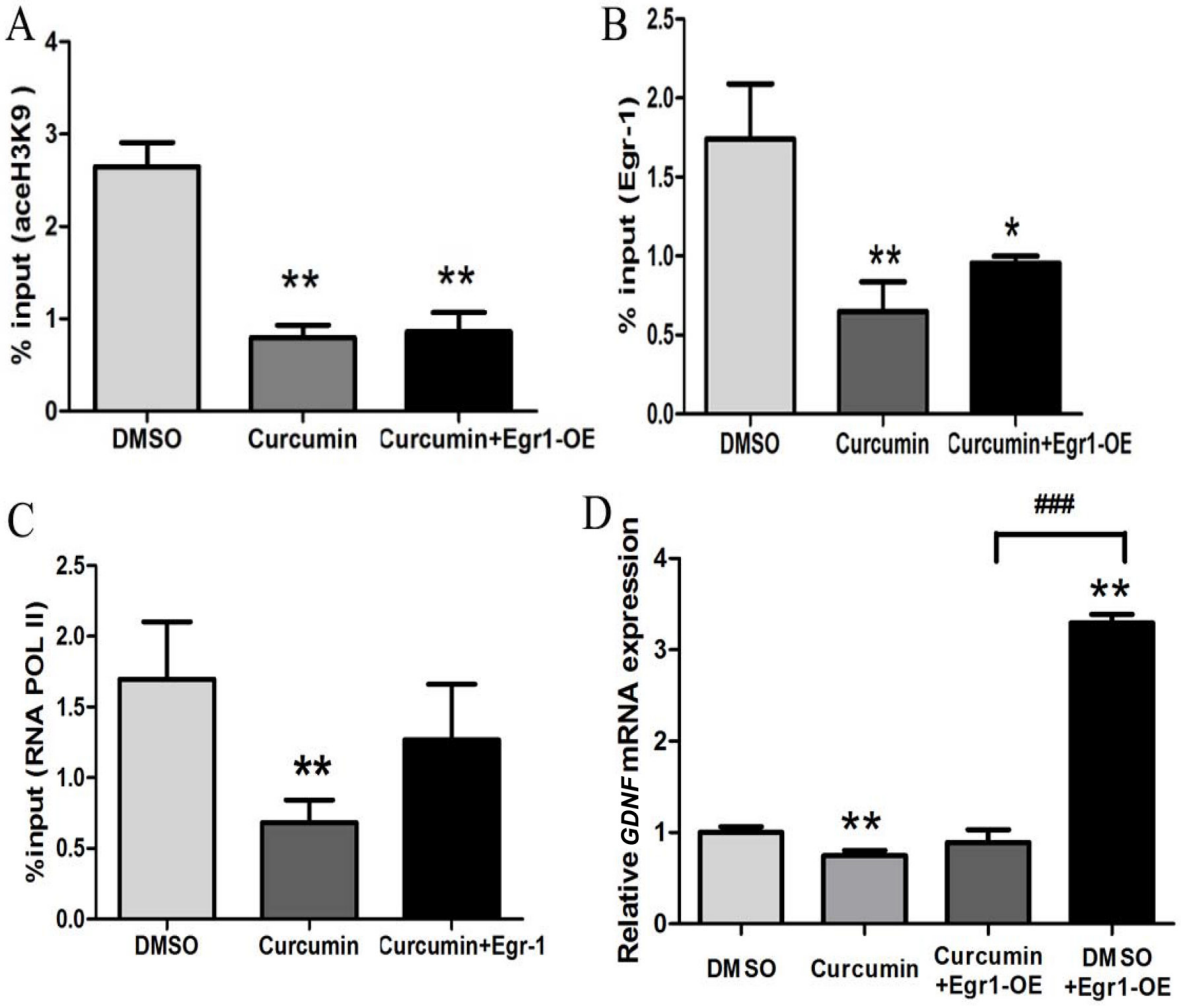
Histone hypoacetylation inhibited *GDNF* transcription induced by Egr-1 overexpression in *GDNF* promoter II in C6 glioma cells Cells in the logarithmic growth phase were inoculated into 6-well plates and treated with 50 μM curcumin or 0.25% DMSO for 24 h when the cells reached 50% confluence. The cells were then infected with 100 MOI of lentivirus Egr 1-OE for 72 h. (**A**) H3K9 acetylation of the Egr-1 binding sites of *GDNF* promoter II in C6 glioma cells was measured using ChIP-PCR. (**B**) Egr-1 binding was measured using ChIP-PCR. (**C**) RNA POL II recruitment in the Egr-1 binding sites of *GDNF* promoter II in C6 glioma cells was measured using ChIP-PCR. (**D**) Relative *GDNF* mRNA expression was measured by real-time PCR. *P < 0.05, **P < 0.01, ^###^*P* < 0.001.

To determine whether histone hyperacetylation in the Egr-1 binding sites of *GDNF* promoter II is necessary for Egr-1 to regulate enhanced *GDNF* transcription in glioma cells, a C6 glioma cell model with histone hypoacetylation in the Egr-1 binding sites of *GDNF* promoter II was established by treatment with curcumin (Figure 6A). The effects of Egr-1 overexpression on histone acetylation, Egr-1 binding to *GDNF* promoter II, RNA POL II recruitment, and *GDNF* transcription in curcumin-treated C6 glioma cells were examined by gene overexpression, ChIP, and real-time PCR studies. The results showed that 24 h after treatment with 50 μM curcumin, H3K9 acetylation in the Egr-1 binding sites of *GDNF* promoter II, Egr-1 binding to *GDNF* promoter II, RNA POL II recruitment in that region, and *GDNF* mRNA expression were significantly decreased compared with the DMSO treatment group (all *P* < 0.01). Moreover, Egr-1 overexpression failed to significantly increase RNA POL II recruitment to the Egr-1 binding sites of *GDNF* promoter II or *GDNF* expression (*P* > 0.05). Conversely, we observed significantly increased *GDNF* transcription in the DMSO treatment group (*P* < 0.001, Figure 6), which suggests that histone hyperacetylation is a prerequisite for highly expressed Egr-1 to regulate *GDNF* transcription. In addition, compared with the curcumin treatment group, Egr-1 overexpression after curcumin treatment did not significantly affect H3K9 acetylation in the Egr-1 binding sites of *GDNF* promoter II (*P* > 0.05), the Egr-1 binding to *GDNF* promoter II (*P* = 0.061), and RNA POL II recruitment in that region (*P* > 0.05, Figure 6A–6C).

### Co-existence but not binding of Egr-1 and RNA POL II in high-grade glioma cell nuclei

**Figure 7:**
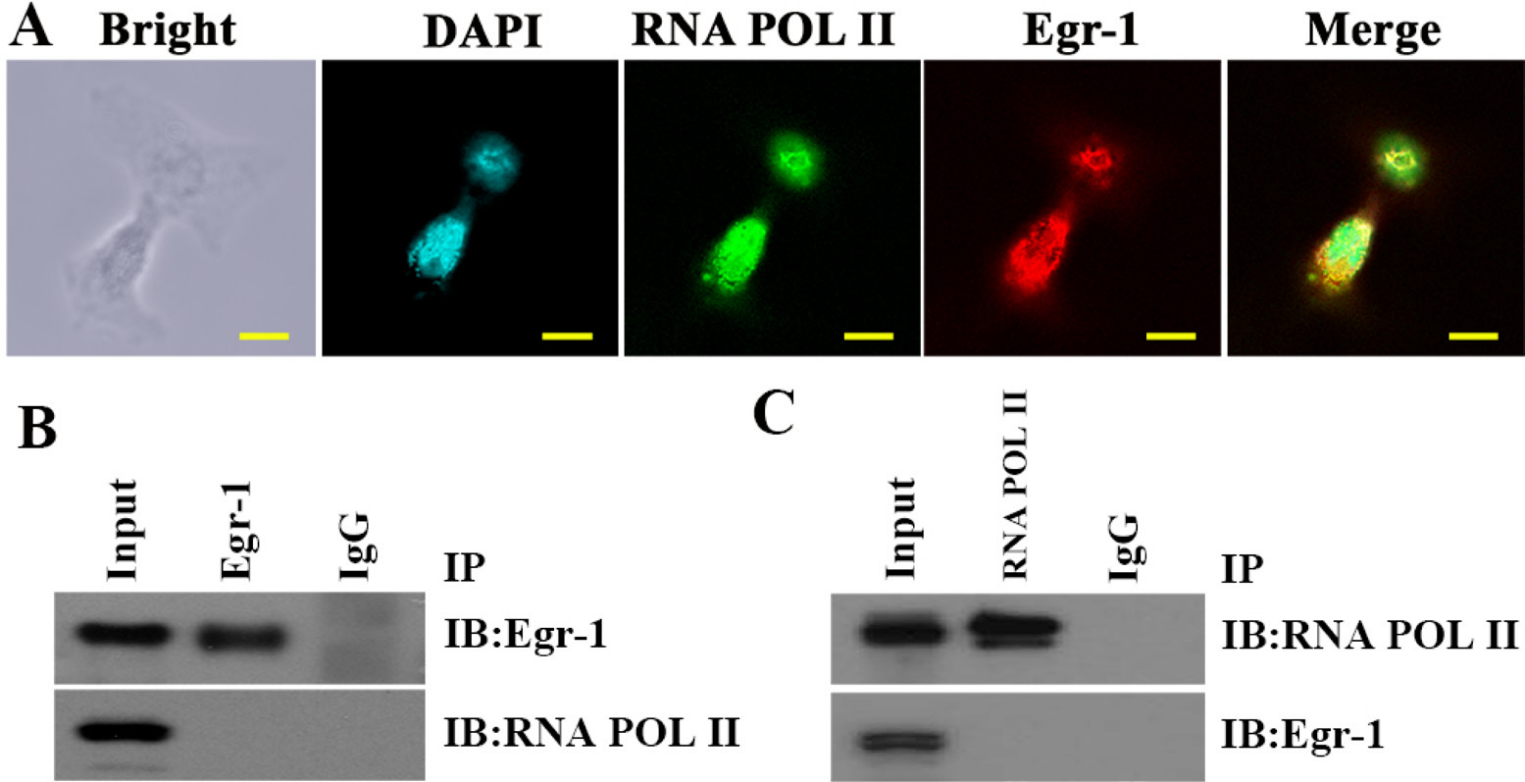
Co-existence but not binding of Egr-1 and RNA POL II in C6 glioma cell nuclei (**A**) Egr-1 and RNA POL II protein locations in C6 glioma cells were determined by immunofluorescence. They were co-expressed in the nuclei of C6 cells with local overlapping regions. The scale is 100 μm. (**B, C**) The binding of Egr-1 to RNA POL II in C6 glioma cell nuclei was measured by co-IP with an Egr-1 antibody followed by western blotting with an RNA polymerase II antibody. Next, the reverse protocol was carried out. The results showed that the proteins were not bound in the nuclei of C6 glioma cells.

To investigate the mode of action between Egr-1 and RNA POL II in high-grade glioma cells, the binding of Egr-1 to RNA POL II in C6 astroglioma cells was measured by double immunofluorescence and co-IP. The results showed that although both Egr-1 and RNA POL II were present in the nuclei of C6 glioma cells with local overlapping regions (Figure 7A), they were not bound to each other (Figure 7B–7C).

## DISCUSSION

Egr-1 is a key transcription factor for *GDNF* gene activation [12, 25, 26] and is involved in histone hyperacetylation-mediated high-level *GDNF* transcription in glioma cells [13]. In this study, H3K9 acetylation in Egr-1 binding sites of *GDNF* promoter II, Egr-1 binding, and RNA POL II recruitment in that region were significantly increased in high-grade glioma tissue. It was therefore hypothesized that Egr-1 and RNA POL II might be synergistically involved in histone hyperacetylation-mediated high-level *GDNF* transcription.

Egr-1 expression was measured in high- and low-grade glioma and normal brain tissue, as well as rat C6 astroglioma cells and normal astrocytes. Compared with the control group, Egr-1 expression was significantly higher in high-grade glioma tissue and C6 astroglioma cells. This is consistent with the high expression of Egr-1 reported by Peng et al. [17, 18] in cancerous tissues such as liver cancer and prostate cancer. In addition, we previously found that *GDNF* transcription was significantly decreased by the knockdown of Egr-1 in C6 glioma cells [13]. This indicates that highly expressed Egr-1 may be involved in regulating the high-level transcription of *GDNF* in high-grade glioma cells.

The effect of Egr-1 overexpression on *GDNF* transcription in glioma cells was examined to test this hypothesis. *GDNF* mRNA expression was significantly increased by Egr-1 overexpression in C6 glioma cells. Moreover, both binding of Egr-1 to *GDNF* promoter II and RNA POL II recruitment in that region were significantly increased under this condition. This is similar to the effect of histone hyperacetylation of the Egr-1 binding sites of *GDNF* promoter II. It has been pointed out that histone acetylation can affect transcription factor regulation [27]. Whether the regulation of *GDNF* induced by Egr-1 overexpression depends on histone hyperacetylation in *GDNF* promoter II was tested in a C6 glioma cell model treated with curcumin as previously described [13]. The results showed that with histone hypoacetylation in the Egr-1 binding sites of *GDNF* promoter II, the binding of Egr-1 and RNA POL II to *GDNF* promoter II or *GDNF* transcription could not be significantly increased, even with Egr-1 overexpression. This indicates that the regulation of *GDNF* induced by overexpressed Egr-1 and RNA POL II in high-grade glioma cells is dependent on histone hyperacetylation of *GDNF* promoter II.

How does Egr-1 interact with RNA POL II under histone hyperacetylation in *GDNF* promoter II? This needed to be further investigated, but it has been suggested that certain gene-specific transcription factors can promote RNA POL II recruitment [28, 29]. It was therefore speculated that Egr-1 might have a similar effect, which led us to examine Egr-1 binding to RNA POL II in C6 glioma cells. The results showed that they co-existed in glioma cell nuclei with local overlapping regions, but the proteins were not bound to each other. This suggests that Egr-1 may be involved in RNA POL II recruitment, but not by direct binding. Egr-1 has three typical zinc finger domains that can bind to the three base pairs in the DNA major groove in the gene promoter. This widens and deepens the DNA major groove, affecting the conformation of nearby DNA molecules [30, 31]. Indeed, our previous studies showed that both histone hyperacetylation [13] and CpG hypermethylation [9] occurred in the Egr-1 binding sites of *GDNF* promoter II in high-grade glioma cells. A recent study found that CpG hypermethylation in the Egr-1 binding motif did not affect its binding to Egr-1 [32]. However, such hypermethylation might inhibit RNA POL II recruitment [27, 33]. Therefore, it was hypothesized that Egr-1 might bind to the DNA major groove in the hypermethylated Egr-1 binding sites of *GDNF* promoter II via its zinc finger domains, deepening the DNA major groove and fully opening the DNA molecules. This would further promote RNA POL II recruitment in that region. Indeed, Egr-1 is able to influence the binding of other factors to the gene promoter by binding to the promoter [34].

In conclusion, we found that H3K9 acetylation in the Egr-1 binding sites of *GDNF* promoter II, binding of Egr-1 to such sites, RNA POL II recruitment in that region, and Egr-1 expression were significantly increased in high-grade glioma tissue. High Egr-1 expression promoted the binding of Egr-1 and RNA POL II to *GDNF* promoter II and enhanced *GDNF* transcription. Furthermore, studies of a C6 glioma cell model with curcumin-induced histone hypoacetylation in *GDNF* promoter II revealed that low acetylation in the Egr-1 binding sites of *GDNF* promoter II significantly decreased Egr-1 binding to *GDNF* promoter II, RNA POL II recruitment, and *GDNF* transcription in C6 glioma cells. These effects could not be reversed by Egr-1 overexpression. In addition, Egr-1 and RNA POL II co-existed in C6 cells but were not bound to each other. Collectively, these findings indicate that in high-grade glioma cells, highly expressed Egr-1 may be involved in recruiting RNA POL II in *GDNF* promoter II in a non-binding manner, suggesting that Egr-1 helps regulate high-level *GDNF* transcription. Furthermore, such regulation is dependent on histone hyperacetylation in *GDNF* promoter II. Our next study will focus on the how Egr-1 and RNA POL II jointly promote high-level *GDNF* transcription in high-grade glioma cells.

## MATERIALS AND METHODS

### Tissue samples

Tissue samples were derived from six patients with acute brain trauma given intracranial decompression and 12 human glioma tissue samples (World Health Organization [WHO] grades I-IV) acquired from the affiliated hospitals of Suzhou University were utilized for ChIP and Western blot assays. Biopsied glioma tissues derived from patients who had not yet undergone cancer therapy were randomly sampled. From these, the specimens with pathological grades I–II were assigned to the low-grade glioma group, while those with pathological grades III–IV were assigned to the high-grade glioma group.

### Cell culture

Cryopreserved rat C6 astroglioma cell line and normal astrocytes were used for the experiments. Cell culture was performed as previously described [11], using medium composed of modified Eagle's medium (Gibco/Invitrogen, Carlsbad, CA, USA) containing 10% FBS (Gibco/Invitrogen), 100 U/mL penicillin, and 100 U/mL streptomycin. All cells were maintained in a 37°C incubator with a 5% CO_2_ humidified atmosphere. Healthy cells derived after two or three passages were chosen for the following experiments.

### Chromatin immunoprecipitation–polymerase chain reaction (ChIP-PCR)

Using the acetyl-histone H3 (Lys9) polyclonal antibody (cat. no.: 07–352, Millipore, Billerica, MA, USA), Egr-1 polyclonal antibody (cat. no.: sc-110X, Santa Cruz Biotechnology [SCBT], Santa Cruz, CA, USA), RNA POL II monoclonal antibody (cat. no.: 05–623, Millipore, Billerica, MA, USA) and normal IgG (cat. no.: sc-2027X, SCBT), ChIP was performed according to the the EZ-chip chromatin immunoprecipitation kit manufacturer's instructions (Millipore, Billerica, MA, USA). Routine polymerase chain reaction (PCR) and real-time PCR were used to detect DNA from immunoprecipitated target antibody. After conventional PCR, the PCR products were detected by agarose gel electrophoresis. After real-time PCR, data quantification was analyzed relative to gene expression data using the 2^−ΔΔCt^ method, and the results were displayed as % Input = 2 ^(CTInput-CTChIP)^ × Input dilution factor × 100. The PCR primer was synthesized by Kangchen Bio-tech Inc. (Shanghai, China), and the sequence is shown in Table 1.

**Table 1:** Primer sequence for ChIP-PCR

Gene name	Primer sequence	Tq (°C)	Primer length (bp)
Hum *GDNF*-P_(−98/+56)_	F:5ʹ CTGCTCGGACCTCGGCTT 3ʹ R:5ʹ GGCAAGAGTTCGCAATCCTG 3ʹ	60	154
Hum *GDNF*–P_(−864/−793)_	F:5ʹ GGCAGCTCCTTTTCTCGC 3ʹ R:5ʹ CTTCCCGCTCGGGTGTCT 3ʹ	60	71
Rat-*GDNF*-P_(−375/−93)_	F:5ʹ CGAGGAGGTGCAGAGTGAGG3ʹ R:5ʹ GGGAGCAAGAGCACGCAA3ʹ	60	283

### Recombinant lentivirus construction and Infection

The full-length rat *Egr-1* cDNA fragment (NM_012551.2) was amplified by PCR technology. The PCR primers were synthesized as follows (Genechem Co., Ltd, Shanghai, China): Egr-1-*AgeI*-F 5′-ATGGACAACTACCCCAAACT-3′, and Egr-1-*AgeI*-R 5′-TTAGCAAATTTCAATTGTCCTAGG-3′. The lentiviral vector Ubi-MCS-GFP (Genechem) was digested by the restriction enzyme *AgeI*. Then the *Egr-1* cDNA fragments were ligated into the lentiviral vector Ubi-MCS-GFP. The primer (5′-AAGCGCTGCTGCCGCTGTTGCT-3′) located in the coding sequence of the *Egr-1* cDNA was used in PCR to identify positive transformants. Positive clones, as confirmed by PCR, were chosen for sequencing. Recombinant lentiviruses, which coexpress enhanced green fluorescent protein (EGFP) and Egr-1 sequence, were produced by 293T cells following the cotransfection of Ubi-*Egr1*-EGFP and the packaging plasmids pHelper1.0 and pHelper2.0 (Genechem). The virus titer was detected by real-time PCR after concentrating and harvesting the viral supernatant. C6 astroglioma cells were plated at a density of 5 × 10^4^ cells/well in a 6-well plate (Corning, NY, USA). After 24 h, cells were infected by 100 MOI concentrated lentivirus in the presence of polybrene (8 μg/ml). 72 h after infection, cells were harvested for the following experiments.

### RNA extraction and real-time PCR

RNA extraction and real-time PCR were performed as previously described [13]. Glyceraldehyde-3-phosphate dehydrogenase (GAPDH) mRNA expression was used as the internal reference for every sample, and the relative mRNA expression levels of the target gene were calculated by relative quantification (2^−ΔΔCT^). The forward and reverse primer sequences for the target gene amplification as well as internal reference gene are shown in Table 2.

**Table 2:** Primer sequence for real-time PCR

Gene name	Primer sequence	Tq (°C)	Primer length (bp)
Hum *Egr-1*	F:5ʹ GGTCAGTGGCCTAGTGAGC3ʹ R:5ʹGTGCCGCTGAGTAAATGGGA3ʹ	59	149
Hum *GAPDH*	F:5ʹGAAGGTGAAGGTCGGAGTC3ʹ F:5ʹGAAGATGGTGATGGGATTTC3ʹ	59	226
Rat *Egr-1*	F:5ʹCACCTGACCACAGAGTCCTT3ʹ R:5ʹCCAGTATAGGTGATGGGAGG3ʹ	59	110
Rat *GDNF*	F:5ʹ GACTTGGGTTTGGGCTACGA3ʹ R:5ʹ TGGTAAACCAGGCTGTCGTC3ʹ	59	209
Rat *GAPDH*	F:5ʹ TCCCTCAAGATTGTCAGCAA3ʹ R:5ʹ AGATCCACAACGGATACATT3ʹ	59	308

### Western blot

Total protein was extracted from each sample with NP-40 lysis buffer. Total extracts were separated on a 10% SDS-PAGE gel and transferred onto NC membrane (Millipore, USA). Blots were blocked using TBST solution containing 5% non-fat milk, and incubated in rabbit anti-mouse Egr-1 polyclonal antibody (1:200, Santa-Cruz, USA) or a mouse anti-avian β-Actin monoclonal antibody (sc-47778, Santa-Cruz, USA). After series of washings with TBST, the NC membrane was incubated in IR-Dye 800cw or 680rd labeled secondary antibodies (1:10,000, LI-COR), and then scanned using a LI-COR Odyssey imaging system (LiCor, Lincoln, NE). β-actin was used as the internal reference protein.

### Immunoprecipitation and immunoblotting

Equal amounts of nuclear protein extracts prepared from C6 cells were incubated with the mouse monoclonal RNA polymerase II antibody (05–623, Millipore, Billerica, MA, USA), rabbit polyclonal Egr-1 antibody (sc-110X, SCBT, Santa Cruz, CA, USA) or normal IgG (sc-2027X, SCBT) overnight at 4°C. Then, the agarose-conjugated protein-A/G beads (SCBT) were added into the immunocomplex and the mixture was incubated at 4°C for another 12 h. After extensive washing with ice-cold WB/IP lysis buffer with protease inhibitors, the beads were mixed with SDS loading buffer and boiled. Immunoblotting were performed as Western Blot described above.

### Immunofluorescence assay

The C6 cells were grown on coverslips in a 24-well plate, fixed with 4% paraformaldehyde (w/v) for 40 min, and permeabilized with 0.5% (w/v) Triton X-100 in PBS for 15 min. The cells were then blocked for 30 min in PBS containing 10% fetal bovine serum (FBS) followed by overnight incubation with the primary antibodies against Egr-1 (1: 250, Santa-Cruz, USA) and RNA polymerase II (1: 300, Millipore, USA). After series of washings with PBS, cells were incubated for 2 h with secondary antibodies (Life, US) conjugated with fluorescein isothiocyanate or tetra methyl rhodamine isothiocyanate (TRITC) in dark moist environment. The coverslips were mounted with hydromount containing DAPI to stain the nuclei (KeyGEN BioTECH, China). The localization of Egr-1 and RNA polymerase II protein was examined using fluorescence confocal microscope (Leica Microsystems).

### Statistical analysis

Statistical analyses were carried out using the SPSS 16.0 software package (SPSS Inc., Chicago, IL, USA) and expressed as mean ± standard deviation (mean ± SD). Independent sample *t* test was used to test the mean value in 2 groups. One-way analysis of variance (ANOVA) was used to determine the significance of any differences between experimental groups. A value of *P* < 0.05 was considered as significantly different.
